# HIV post exposure prophylaxis induced bicytopenia: a case report

**DOI:** 10.1186/1742-6405-11-11

**Published:** 2014-02-07

**Authors:** Benjamin T Schleenvoigt, John P Fobiwe, Peter M Keller, Stefan Hagel, Mathias W Pletz

**Affiliations:** 1 Center for Infectious Diseases and Infection Control, Jena University Hospital, Erlanger Allee 101, Jena, Thuringia 07740, Germany; 2 Department of Internal Medicine IV, Jena University Hospital, Jena, Germany; 3 Institute for Medical Microbiology, Jena University Hospital, Jena, Germany; 4 Center for Sepsis Control and Care (CSCC), Jena University Hospital, Jena, Germany

**Keywords:** HIV, Post exposure prophylaxis, Leukopenia, Thrombocytopenia, Tenofovir, Emtricitabin, Lopinavir, Ritonavir

## Abstract

Long and short term side effects of antiretroviral drugs are not fully understood yet. Here a case of reversible blood count changes following post exposure prophylaxis with tenofovir/emtricitabin and lopinavir/ritonavir is reported. We propose that antiretroviral drugs used in post exposure prophylaxis may have a significant impact on hematopoiesis.

## Background

Needle prick injury is common in clinical practice. In order to prevent HIV infection, post exposure prophylaxis (PEP) is considered in situations with potential risk of infection [1,2]. Long and short term side effects of the drugs used are not fully understood yet. Here, we report a case of reversible leukopenia and thrombocytopenia following a 28 days course of post exposure prophylaxis with tenofovir/emtricitabin and lopinavir/ritonavir.

## Case presentation

A 56 years old male patient presented after occupational needle prick injury. Index patient could not be determined and PEP was started within 28 hours with lopinavir 400 mg/ritonavir 100 mg BD and tenofovir 245 mg/emtricitabin 200 mg QD and was continued for 28 days. Serology for HIV, HCV, HBV as well as parameters for blood count (leukocytes 4.6 Gpt/l, thrombocytes 146 Gpt/l), liver and renal function tests were unremarkable. Hepatitis B vaccination was administered. Past medical history revealed coronary heart disease, hypertension and the patient reported known marginal reduction of platelets in absence of any hemic disease. The concurrent medication consisted of ramipril 5 mg QD, acetylsalicylacid 100 mg QD and simvastatin 40 mg QD. The statin was paused during PEP.

Antiretroviral post exposure treatment was clinically well tolerated and the patient reported no symptoms of rash or gastrointestinal side effects. Control of laboratory parameters on day 19 after initiation of PEP showed a slight decrease in WBC to 4.0 Gpt/l. Investigation on day 33 (5 days after the end of PEP) showed bicytopenia with leukopenia 2.0 Gpt/l and thrombocytopenia 97 Gpt/l. A second control on day 40 revealed a return to a normal blood count and no alterations of differential blood count (neutrophil granulocytes 3.61 Gpt/l, lymphocytes 1.46 Gpt/l, monocytes 0.39 Gpt/l, eosinophil granulocytes 0.06 Gpt/l, basophil granulocytes 0.02 Gpt/l). Serum electrophoresis was unremarkable (total protein 70.4 g/l, albumin 66.9%, alpha-1-globulin 3.6%, alpha-2-globulin 8.4%, beta-globulin 9.7%, gamma-globulin 11.4%) and determination of ANA and pANCA as well as folic acid and vitamin B 12 levels revealed normal values. There was no evidence of blood count changes during follow up over 6 months and the patient remained sero-negative for HIV and HCV. After stratification of benefits and risks, no further invasive clarification of pathogenicity was initiated.

## Conclusions

Cytopenia such as anemia, thrombocytopenia, neutropenia or lymphopenia is a known effect of HIV and AIDS status is an identified risk factor. A recent Korean publication pointed out the impact of HIV alone on hematologic manifestations. In this study cytopenia was shown to be reversible with antiretroviral treatment [3]. Thrombocytopenia or leukopenia following antiretroviral post exposure therapy with tenofovir or emtricitabin have not been described yet. In a retrospective study leukopenia was associated with lopinavir/ritonavir [4]. A thorough review revealed a single case of thrombocytopenia associated with lopinavir/ritonavir [5]. As a mechanism of pathogenicity autoimmune causes can be discussed for thrombocytopenia in our case. Since ANA and ANCA were tested negative this hypothesis is less convincible. As leukopenia emerged simultaneously to thrombocytopenia a direct impact on hematopoiesis seems more plausible. Thrombocytopenia was described for other protease inhibitors such as saquinavir, but to this date no feasible hypothesis for pathogenicity is available [6]. Based on previous observations and this case report we propose that antiretroviral drugs used in PEP may have a direct impact on hematopoiesis. The precise mechanism should be further investigated.

## Consent

Written informed consent was obtained from the patient for publication of this case report. A copy of the written consent is available for review by the Editor-in-Chief of this journal.

## Abbreviations

AIDS: Acquired immunodeficiency syndrome; ANA: Anti-nuclear antibody; ANCA: Anti-neutrophil cytoplasmic antibody; HBV: Hepatitis B virus; HCV: Hepatitis C virus; HIV: Human immunodeficiency virus; PEP: Post exposure prophylaxis; WBC: White blood cells.

## Competing interests

The authors declare that they have no competing interests.

## Authors’ contributions

The patient was treated and followed up by BTS and JPF. BTS conceived the case report, did the literature research and drafted the manuscript. It was critically corrected by PMK and SH. MWP helped to draft the manuscript and participated in the literature research and coordination. All authors read and approved the final manuscript.

## References

[B1] Gemeinsame Erklarung der Deutschen A-G Post-exposure prophylaxis of HIV infection. German-Austrian recommendations, update September 2007 Dtsch Med Wochenschr 2009 11 Suppl 1 S16 33 19172552 10.1055/s-0028-1123966

[B2] Guidelines Version 7.0 October 2013 http://www.eacsociety.org

[B3] Choi SY Kim I Kim NJ Lee SA Choi YA Bae JY Kwon JH Choe PG Park WB Yoon SS Hematological manifestations of human immunodeficiency virus infection and the effect of highly active anti-retroviral therapy on cytopenia Korean J Hematol 2011 11 253 257 10.5045/kjh.2011.46.4.253 22259631 PMC3259517

[B4] Alp E Bozkurt I Doganay M Epidemiological and clinical characteristics of HIV/AIDS patients followed-up in Cappadocia region: 18 years experience Mikrobiyol Bul 2011 11 125 136 21341167

[B5] Colebunders R De Schacht C Vanwolleghem T Callens S Lopinavir/ritonavir- and indinavir-induced thrombocytopenia in a patient with HIV infection Int J Infect Dis 2004 11 315 316 10.1016/j.ijid.2004.05.001 15325601

[B6] Fellay J Boubaker K Ledergerber B Bernasconi E Furrer H Battegay M Hirschel B Vernazza P Francioli P Greub G Prevalence of adverse events associated with potent antiretroviral treatment: swiss HIV cohort study Lancet 2001 11 1322 1327 10.1016/S0140-6736(01)06413-3 11684213

